# Diethyl 4-(1*H*-imidazol-2-yl)-2,6-dimethyl-1,4-di­hydro­pyridine-3,5-di­carboxyl­ate

**DOI:** 10.1107/S2414314620000346

**Published:** 2020-01-17

**Authors:** Miri Yoo, Dongsoo Koh

**Affiliations:** a Department of Applied Chemistry, Dongduk Women’s University, Seoul 136-714, Republic of Korea; Goethe-Universität Frankfurt, Germany

**Keywords:** crystal structure, 1,4-di­hydro­pyridine, N—H⋯O hydrogen bonds, N—H⋯N hydrogen bonds

## Abstract

In the title compound, C_16_H_21_N_3_O_4_, the 1,4-di­hydro­pyridine ring adopts a flattened boat conformation, with the imidazole substituent in an axial orientation [dihedral angle between ring planes = 82.9 (6)°]. In the crystal structure, pairs of N—H⋯O and N—H⋯N hydrogen bonds with graph-set notation *R*_2_^2^(14) connect the mol­ecules into chains running along the *c*-axis direction.

## Abstract 2

The crystal structure of a biologically important pharmacophore containing 1,4-di­hydro­pyridine is reported.

## Graphical abstract

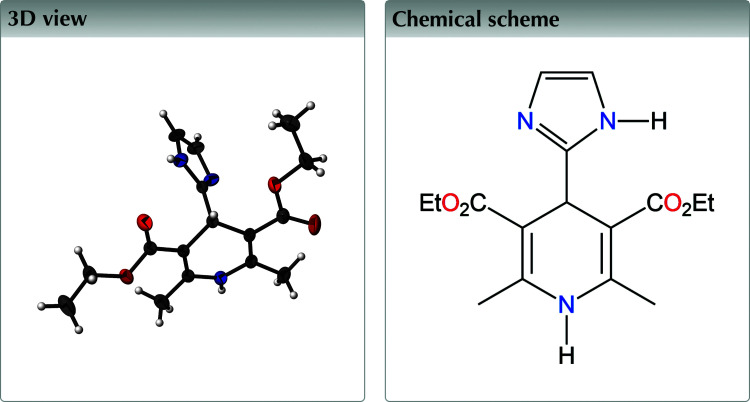

## Structure description

**Figure 1:**
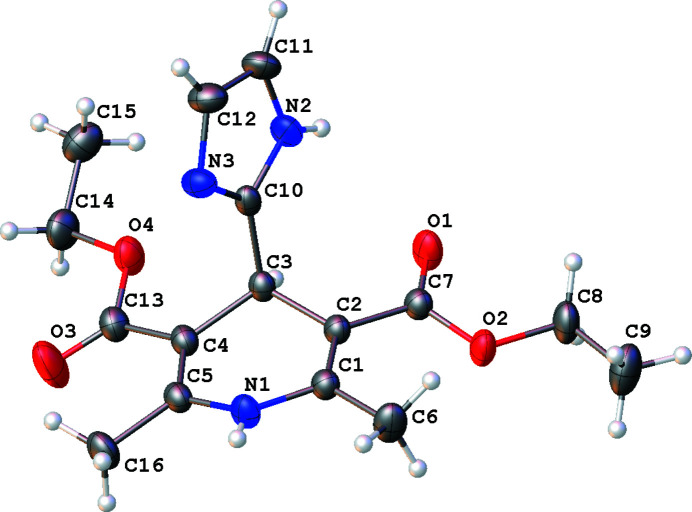
The mol­ecular structure of the title compound, showing the atom-labelling scheme with displacement ellipsoids drawn at the 30% probability level.

Hantzsch 1,4-di­hydro­pyridines (1,4-DHPs) have shown broad biological activities which include calcium channel blocker (Schaller *et al.*, 2018 ▸), anti­mycobacterial (Lentz *et al.*, 2016 ▸), anti­convulsant (Prasanthi *et al.*, 2014 ▸) and anti-tubercular (Khoshneviszadeh *et al.*, 2009 ▸) activities. According to our recent report, they show anti-cancer activities in HCT116 human colon cancer cell lines (Ahn *et al.*, 2018 ▸). We report herein the synthesis and crystal structure of the title compound (Fig. 1 ▸).

**Figure 2:**
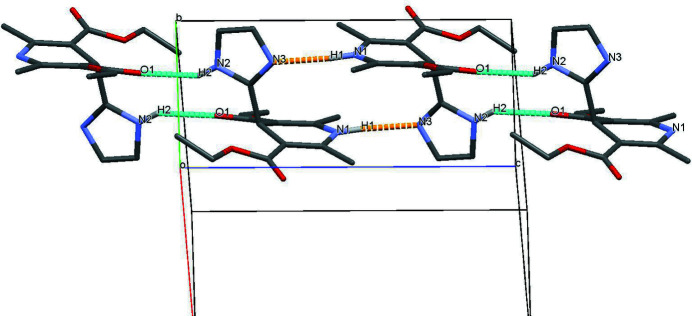
Part of the crystal structure with two inter­mol­ecular hydrogen bonds (blue and yellow dashed lines) are shown. For clarity, only those H atoms involved in hydrogen bonding are shown.

In the title compound, the 1,4-di­hydro­pyridine (C1–C5/N1) ring is twisted slightly from planarity, with a maximum deviation of 0.178 (1) Å at C3 (r.m.s. deviation = 0.113 Å). The dihedral angle formed between the plane of the 1,4-di­hydro­pyridine (C1–C5/N1) and imidazole (C10–C12/N2–N3) rings is 82.9 (6)°. One of the carbonyl groups (C13=O3) lies on the same side as the methyl group at C16 with the other carbonyl group (C7=O1) on the opposite side. In the crystal, pairs of N—H⋯O and N—H⋯N hydrogen bonds with graph-set notation 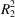 (14) connect the mol­ecules into chains running along the *c*-axis direction (Table 1 ▸, Fig. 2 ▸).

## Synthesis and crystallization

Methyl aceto­acetate (20 mmol) and 1*H*-imidazole-2-carbaldehyde (10 mmol) were dissolved in 30 ml of ethanol to give a clear solution. To the mixture, ammonium acetate (10 mmol) was added and the reaction mixture was heated at 365 K for 5 h. After completion of the reaction (monitored by TLC), the mixture was cooled to room temperature to produce a solid product. This solid was recrystallized from ethanol solution to obtain single-crystal of the title compound in 61% yield.

## Refinement

Crystal data, data collection and structure refinement details are summarized in Table 2 ▸.

## Supplementary Material

Crystal structure: contains datablock(s) I. DOI: 10.1107/S2414314620000346/bt4088sup1.cif

Structure factors: contains datablock(s) I. DOI: 10.1107/S2414314620000346/bt4088Isup2.hkl

Supporting information file. DOI: 10.1107/S2414314620000346/bt4088Isup3.cml

CCDC reference: 1977288

Additional supporting information:  crystallographic information; 3D view; checkCIF report

## Figures and Tables

**Table 1:** Hydrogen-bond geometry (Å, °)

*D*—H⋯*A*	*D*—H	H⋯*A*	*D*⋯*A*	*D*—H⋯*A*
N1—H1⋯N3^i^	0.87	2.05	2.913 (3)	175
N2—H2⋯O1^ii^	0.87	2.18	2.979 (3)	152

Symmetry codes: (i) 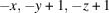 ; (ii) 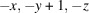 .

**Table 2:** Experimental details

Crystal data	
Chemical formula	C_16_H_21_N_3_O_4_
*M*_r_	319.36
Crystal system, space group	Triclinic, *P* 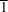
Temperature (K)	223
*a*, *b*, *c* (Å)	8.127 (7), 8.411 (9), 12.536 (10)
α, β, γ (°)	105.36 (4), 96.52 (2), 94.77 (3)
*V* (Å^3^)	815.1 (13)
*Z*	2
Radiation type	Mo *K*α
μ (mm^−1^)	0.10
Crystal size (mm)	0.28 × 0.21 × 0.14

Data collection	
Diffractometer	PHOTON 100 CMOS
Absorption correction	Multi-scan (*SADABS*; Bruker, 2012 ▸)
*T*_min_, *T*_max_	0.974, 0.987
No. of measured, independent and observed [*I* > 2σ(*I*)] reflections	33431, 3920, 3144
*R*_int_	0.041
(sin θ/λ)_max_ (Å^−1^)	0.667

Refinement	
*R*[*F* ^2^ > 2σ(*F* ^2^)], *wR*(*F* ^2^), *S*	0.049, 0.142, 1.06
No. of reflections	3920
No. of parameters	212
H-atom treatment	H-atom parameters constrained
Δρ_max_, Δρ_min_ (e Å^−3^)	0.32, −0.23

Computer programs: *APEX2* and *SAINT* (Bruker, 2012 ▸), *SHELXS97* (Sheldrick, 2008 ▸), *SHELXL2014* (Sheldrick, 2015 ▸), *SHELXTL* (Sheldrick, 2008 ▸) and *publCIF* (Westrip, 2010 ▸).

## full crystallographic data

### Crystal data

**Table:**

C_16_H_21_N_3_O_4_	*Z* = 2
*M**_r_* = 319.36	*F*(000) = 340
Triclinic, *P*1	*D*_x_ = 1.301 Mg m^−^^3^
*a* = 8.127 (7) Å	Mo *K*α radiation, λ = 0.71073 Å
*b* = 8.411 (9) Å	Cell parameters from 9982 reflections
*c* = 12.536 (10) Å	θ = 2.5–28.3°
α = 105.36 (4)°	µ = 0.10 mm^−^^1^
β = 96.52 (2)°	*T* = 223 K
γ = 94.77 (3)°	Block, colourless
*V* = 815.1 (13) Å^3^	0.28 × 0.21 × 0.14 mm

### Data collection

**Table:**

PHOTON 100 CMOS diffractometer	3144 reflections with *I* > 2σ(*I*)
φ and ω scans	*R*_int_ = 0.041
Absorption correction: multi-scan (SADABS; Bruker, 2012)	θ_max_ = 28.3°, θ_min_ = 2.5°
*T*_min_ = 0.974, *T*_max_ = 0.987	*h* = −10→10
33431 measured reflections	*k* = −11→11
3920 independent reflections	*l* = −16→16

### Refinement

**Table:**

Refinement on *F*^2^	0 restraints
Least-squares matrix: full	Hydrogen site location: inferred from neighbouring sites
*R*[*F*^2^ > 2σ(*F*^2^)] = 0.049	H-atom parameters constrained
*wR*(*F*^2^) = 0.142	*w* = 1/[σ^2^(*F*_o_^2^) + (0.0734*P*)^2^ + 0.2436*P*] where *P* = (*F*_o_^2^ + 2*F*_c_^2^)/3
*S* = 1.06	(Δ/σ)_max_ < 0.001
3920 reflections	Δρ_max_ = 0.32 e Å^−^^3^
212 parameters	Δρ_min_ = −0.23 e Å^−^^3^

### Special details

**Table:**

Geometry. All esds (except the esd in the dihedral angle between two l.s. planes) are estimated using the full covariance matrix. The cell esds are taken into account individually in the estimation of esds in distances, angles and torsion angles; correlations between esds in cell parameters are only used when they are defined by crystal symmetry. An approximate (isotropic) treatment of cell esds is used for estimating esds involving l.s. planes.

### Fractional atomic coordinates and isotropic or equivalent isotropic displacement parameters (Å^2^)

**Table:**

	*x*	*y*	*z*	*U*_iso_*/*U*_eq_	
N1	0.18302 (15)	0.47356 (14)	0.46454 (9)	0.0280 (3)	
H1	0.1979	0.5159	0.5367	0.034*	
C1	0.21728 (16)	0.57673 (16)	0.39890 (11)	0.0251 (3)	
C2	0.16729 (15)	0.52079 (16)	0.28644 (10)	0.0232 (3)	
C3	0.04796 (16)	0.36033 (15)	0.23758 (10)	0.0229 (3)	
H3	0.0781	0.3040	0.1637	0.028*	
C4	0.06773 (16)	0.24414 (16)	0.31144 (11)	0.0253 (3)	
C5	0.12622 (16)	0.30616 (17)	0.42189 (11)	0.0261 (3)	
C6	0.3103 (2)	0.74263 (19)	0.46493 (12)	0.0374 (4)	
H6A	0.4280	0.7424	0.4580	0.056*	
H6B	0.2959	0.7626	0.5430	0.056*	
H6C	0.2669	0.8296	0.4363	0.056*	
C7	0.21403 (16)	0.60658 (17)	0.20487 (11)	0.0258 (3)	
O1	0.15784 (14)	0.55990 (13)	0.10544 (8)	0.0368 (3)	
O2	0.32952 (13)	0.73897 (13)	0.24786 (8)	0.0333 (3)	
C8	0.3728 (2)	0.83898 (19)	0.17506 (13)	0.0387 (4)	
H8A	0.2719	0.8672	0.1369	0.046*	
H8B	0.4361	0.7785	0.1186	0.046*	
C9	0.4771 (3)	0.9939 (2)	0.24842 (16)	0.0521 (5)	
H9A	0.4123	1.0534	0.3030	0.078*	
H9B	0.5110	1.0643	0.2028	0.078*	
H9C	0.5754	0.9640	0.2867	0.078*	
C10	−0.13059 (16)	0.39732 (15)	0.21997 (10)	0.0231 (3)	
N2	−0.20049 (15)	0.42932 (16)	0.12553 (10)	0.0315 (3)	
H2	−0.1525	0.4293	0.0669	0.038*	
C11	−0.36104 (19)	0.4617 (2)	0.13918 (13)	0.0384 (4)	
H11	−0.4397	0.4889	0.0878	0.046*	
C12	−0.38247 (19)	0.4467 (2)	0.24119 (13)	0.0373 (3)	
H12	−0.4814	0.4613	0.2730	0.045*	
N3	−0.23804 (14)	0.40668 (16)	0.29244 (9)	0.0302 (3)	
C13	0.00797 (18)	0.06611 (17)	0.26232 (12)	0.0308 (3)	
O3	0.02408 (19)	−0.04606 (14)	0.30548 (11)	0.0556 (4)	
O4	−0.07121 (14)	0.03801 (12)	0.15720 (9)	0.0360 (3)	
C14	−0.1454 (2)	−0.13100 (19)	0.10123 (14)	0.0406 (4)	
H14A	−0.2060	−0.1768	0.1517	0.049*	
H14B	−0.0586	−0.2015	0.0776	0.049*	
C15	−0.2623 (2)	−0.1240 (2)	0.00188 (14)	0.0441 (4)	
H15A	−0.3471	−0.0535	0.0264	0.066*	
H15B	−0.3149	−0.2350	−0.0377	0.066*	
H15C	−0.2006	−0.0790	−0.0475	0.066*	
C16	0.1393 (2)	0.2112 (2)	0.50829 (13)	0.0372 (4)	
H16A	0.0529	0.1174	0.4870	0.056*	
H16B	0.1256	0.2835	0.5803	0.056*	
H16C	0.2479	0.1716	0.5132	0.056*	

### Atomic displacement parameters (Å^2^)

**Table:**

	*U*^11^	*U*^22^	*U*^33^	*U*^12^	*U*^13^	*U*^23^
N1	0.0361 (6)	0.0299 (6)	0.0185 (5)	0.0004 (5)	0.0041 (4)	0.0086 (4)
C1	0.0256 (6)	0.0272 (6)	0.0237 (6)	0.0016 (5)	0.0050 (5)	0.0090 (5)
C2	0.0240 (6)	0.0247 (6)	0.0222 (6)	0.0008 (5)	0.0045 (5)	0.0088 (5)
C3	0.0280 (6)	0.0228 (6)	0.0186 (6)	0.0010 (5)	0.0044 (5)	0.0068 (4)
C4	0.0273 (6)	0.0246 (6)	0.0269 (6)	0.0043 (5)	0.0060 (5)	0.0111 (5)
C5	0.0268 (6)	0.0288 (7)	0.0265 (6)	0.0045 (5)	0.0061 (5)	0.0130 (5)
C6	0.0467 (9)	0.0365 (8)	0.0239 (7)	−0.0088 (7)	0.0006 (6)	0.0055 (6)
C7	0.0272 (6)	0.0277 (6)	0.0236 (6)	0.0010 (5)	0.0049 (5)	0.0092 (5)
O1	0.0488 (6)	0.0388 (6)	0.0221 (5)	−0.0082 (5)	0.0029 (4)	0.0124 (4)
O2	0.0368 (6)	0.0343 (5)	0.0292 (5)	−0.0092 (4)	0.0029 (4)	0.0144 (4)
C8	0.0481 (9)	0.0347 (8)	0.0352 (8)	−0.0084 (7)	0.0090 (7)	0.0158 (6)
C9	0.0616 (12)	0.0394 (9)	0.0509 (10)	−0.0158 (8)	0.0143 (9)	0.0092 (8)
C10	0.0296 (6)	0.0211 (6)	0.0170 (6)	−0.0020 (5)	0.0001 (5)	0.0055 (4)
N2	0.0356 (6)	0.0397 (7)	0.0216 (6)	0.0011 (5)	0.0005 (4)	0.0147 (5)
C11	0.0332 (8)	0.0492 (9)	0.0336 (8)	0.0043 (7)	−0.0072 (6)	0.0180 (7)
C12	0.0272 (7)	0.0506 (9)	0.0353 (8)	0.0071 (6)	0.0013 (6)	0.0144 (7)
N3	0.0274 (6)	0.0424 (7)	0.0228 (6)	0.0058 (5)	0.0034 (4)	0.0119 (5)
C13	0.0350 (7)	0.0270 (7)	0.0325 (7)	0.0034 (5)	0.0064 (6)	0.0113 (5)
O3	0.0870 (10)	0.0298 (6)	0.0494 (7)	−0.0010 (6)	−0.0086 (7)	0.0200 (5)
O4	0.0481 (6)	0.0242 (5)	0.0332 (6)	−0.0033 (4)	0.0001 (5)	0.0083 (4)
C14	0.0466 (9)	0.0245 (7)	0.0461 (9)	−0.0043 (6)	0.0013 (7)	0.0065 (6)
C15	0.0401 (9)	0.0456 (9)	0.0402 (9)	−0.0039 (7)	0.0046 (7)	0.0040 (7)
C16	0.0491 (9)	0.0376 (8)	0.0299 (7)	0.0046 (7)	0.0038 (6)	0.0187 (6)

### Geometric parameters (Å, º)

**Table:**

N1—C1	1.3777 (19)	C9—H9B	0.9700
N1—C5	1.385 (2)	C9—H9C	0.9700
N1—H1	0.8700	C10—N3	1.3221 (19)
C1—C2	1.364 (2)	C10—N2	1.3558 (19)
C1—C6	1.508 (2)	N2—C11	1.375 (2)
C2—C7	1.464 (2)	N2—H2	0.8700
C2—C3	1.532 (2)	C11—C12	1.347 (2)
C3—C10	1.513 (2)	C11—H11	0.9400
C3—C4	1.520 (2)	C12—N3	1.383 (2)
C3—H3	0.9900	C12—H12	0.9400
C4—C5	1.357 (2)	C13—O3	1.214 (2)
C4—C13	1.476 (2)	C13—O4	1.350 (2)
C5—C16	1.506 (2)	O4—C14	1.452 (2)
C6—H6A	0.9700	C14—C15	1.496 (3)
C6—H6B	0.9700	C14—H14A	0.9800
C6—H6C	0.9700	C14—H14B	0.9800
C7—O1	1.2239 (19)	C15—H15A	0.9700
C7—O2	1.3434 (19)	C15—H15B	0.9700
O2—C8	1.4473 (19)	C15—H15C	0.9700
C8—C9	1.506 (2)	C16—H16A	0.9700
C8—H8A	0.9800	C16—H16B	0.9700
C8—H8B	0.9800	C16—H16C	0.9700
C9—H9A	0.9700		
			
C1—N1—C5	123.55 (12)	H9A—C9—H9B	109.5
C1—N1—H1	118.2	C8—C9—H9C	109.5
C5—N1—H1	118.2	H9A—C9—H9C	109.5
C2—C1—N1	118.86 (13)	H9B—C9—H9C	109.5
C2—C1—C6	128.14 (12)	N3—C10—N2	110.72 (13)
N1—C1—C6	113.00 (13)	N3—C10—C3	126.17 (12)
C1—C2—C7	124.90 (13)	N2—C10—C3	123.10 (12)
C1—C2—C3	120.01 (11)	C10—N2—C11	107.57 (12)
C7—C2—C3	115.05 (12)	C10—N2—H2	126.2
C10—C3—C4	111.44 (10)	C11—N2—H2	126.2
C10—C3—C2	111.01 (12)	C12—C11—N2	105.92 (13)
C4—C3—C2	110.65 (12)	C12—C11—H11	127.0
C10—C3—H3	107.9	N2—C11—H11	127.0
C4—C3—H3	107.9	C11—C12—N3	110.26 (14)
C2—C3—H3	107.9	C11—C12—H12	124.9
C5—C4—C13	121.28 (13)	N3—C12—H12	124.9
C5—C4—C3	120.13 (13)	C10—N3—C12	105.53 (13)
C13—C4—C3	118.36 (13)	O3—C13—O4	121.56 (14)
C4—C5—N1	119.54 (12)	O3—C13—C4	127.60 (15)
C4—C5—C16	126.95 (14)	O4—C13—C4	110.84 (12)
N1—C5—C16	113.51 (13)	C13—O4—C14	116.62 (12)
C1—C6—H6A	109.5	O4—C14—C15	106.98 (14)
C1—C6—H6B	109.5	O4—C14—H14A	110.3
H6A—C6—H6B	109.5	C15—C14—H14A	110.3
C1—C6—H6C	109.5	O4—C14—H14B	110.3
H6A—C6—H6C	109.5	C15—C14—H14B	110.3
H6B—C6—H6C	109.5	H14A—C14—H14B	108.6
O1—C7—O2	122.36 (12)	C14—C15—H15A	109.5
O1—C7—C2	123.54 (13)	C14—C15—H15B	109.5
O2—C7—C2	114.06 (12)	H15A—C15—H15B	109.5
C7—O2—C8	117.63 (12)	C14—C15—H15C	109.5
O2—C8—C9	106.51 (14)	H15A—C15—H15C	109.5
O2—C8—H8A	110.4	H15B—C15—H15C	109.5
C9—C8—H8A	110.4	C5—C16—H16A	109.5
O2—C8—H8B	110.4	C5—C16—H16B	109.5
C9—C8—H8B	110.4	H16A—C16—H16B	109.5
H8A—C8—H8B	108.6	C5—C16—H16C	109.5
C8—C9—H9A	109.5	H16A—C16—H16C	109.5
C8—C9—H9B	109.5	H16B—C16—H16C	109.5
			
C5—N1—C1—C2	11.5 (2)	C3—C2—C7—O2	−174.61 (11)
C5—N1—C1—C6	−168.01 (13)	O1—C7—O2—C8	7.2 (2)
N1—C1—C2—C7	−171.32 (12)	C2—C7—O2—C8	−175.03 (12)
C6—C1—C2—C7	8.1 (2)	C7—O2—C8—C9	169.35 (14)
N1—C1—C2—C3	11.18 (19)	C4—C3—C10—N3	32.28 (17)
C6—C1—C2—C3	−169.38 (13)	C2—C3—C10—N3	−91.54 (16)
C1—C2—C3—C10	96.34 (14)	C4—C3—C10—N2	−149.20 (12)
C7—C2—C3—C10	−81.40 (14)	C2—C3—C10—N2	86.98 (15)
C1—C2—C3—C4	−27.93 (17)	N3—C10—N2—C11	0.37 (16)
C7—C2—C3—C4	154.33 (11)	C3—C10—N2—C11	−178.36 (12)
C10—C3—C4—C5	−98.67 (15)	C10—N2—C11—C12	−0.51 (17)
C2—C3—C4—C5	25.35 (17)	N2—C11—C12—N3	0.48 (19)
C10—C3—C4—C13	75.95 (15)	N2—C10—N3—C12	−0.07 (16)
C2—C3—C4—C13	−160.02 (12)	C3—C10—N3—C12	178.60 (12)
C13—C4—C5—N1	179.24 (12)	C11—C12—N3—C10	−0.26 (18)
C3—C4—C5—N1	−6.29 (19)	C5—C4—C13—O3	−11.3 (2)
C13—C4—C5—C16	−0.2 (2)	C3—C4—C13—O3	174.13 (16)
C3—C4—C5—C16	174.29 (13)	C5—C4—C13—O4	169.00 (12)
C1—N1—C5—C4	−14.2 (2)	C3—C4—C13—O4	−5.56 (17)
C1—N1—C5—C16	165.30 (13)	O3—C13—O4—C14	3.9 (2)
C1—C2—C7—O1	−174.51 (14)	C4—C13—O4—C14	−176.41 (12)
C3—C2—C7—O1	3.1 (2)	C13—O4—C14—C15	165.03 (13)
C1—C2—C7—O2	7.8 (2)		

### Hydrogen-bond geometry (Å, º)

**Table:**

*D*—H···*A*	*D*—H	H···*A*	*D*···*A*	*D*—H···*A*
N1—H1···N3^i^	0.87	2.05	2.913 (3)	175
N2—H2···O1^ii^	0.87	2.18	2.979 (3)	152

Symmetry codes: (i) −*x*, −*y*+1, −*z*+1; (ii) −*x*, −*y*+1, −*z*.
